# Restaurants

**Published:** 1875-08

**Authors:** 


### RESTAURANTS.

It sometimes falls to the lot of everybody, we presume, to patronise restaurants. They are a great convenience and would be a great acquisition to the world, if they only acted as restore-ants should. But much of the food which they set out is not calculated to restore, is inadequate to sustain. If you take a seat at the table of one of these eating places, you will be puzzled what to call for. The bill of fare will announce meats and vegetables and pastry, little else. The meats are likely to be poor and tough and muscular naturally, and the kind of cooking to which they are subjected, makes them absolutely dangerous to swallow. The nutritive value of meat rests not at all in its solid parts, but solely in its juices. If it have no juice, then it is worthless as food. If you chew a fragment of meat long enough to extract all the juices, and then eject the remainder from your mouth, you have lost nothing but bulk, and bulk, however important, is far better obtained from something besides tough muscular fibres. In nine cases out of ten, it is tough muscle which comes to you in response to your call for beef-steak, and the same is ordinarily true of roast beef and corned beef. The latter has had its juices extracted by the salt and no great amount of nourishment remains. As found on most tables, it is too little boiled to be fit to put in the stomach.

Your vegetables will mostly be underdone. Peas and beans will *never* be done enough; not once in a million times are these valuable and nutritive vegetables sufficiently cooked, even in the houses of the best people. As for dried peas and beans,—beyond question the best of food if properly treated—they are positively dangerous as ordinarily served. They should be stewed for a day or two, preparatory to being baked for some hours, if it is expected to destroy their bad qualities and render them the most wholesome of eatables.

Your restaurant potato is an abomination. To keep it warm it is kept in warm water, and so becomes "water-logged," soggy and worthless. Of course the water should be drained off just before the potatoes are done, and afterwards they should be exposed to dry heat until all moisture is evolved and they become a bunch of starch. Potatoes are not powerful food at any time, and science shows that persons who live on them have very brittle bones; but they have a useful place, and should not be rendered worse than useless by bad treatment.

What we have said of one or two meats and vegetables is true in the main of all. Little is left us, then, but pastry, and this is abominable. Of course, to be what is called "good," it must be greasy, and it would be hard to tell what sort of grease enters into it. In these days when "choice butter" is made from soap-fat and tallow-chandler's stock, it is difficult to say what sort of stuff "cooking butter" is, or from what doubtful source it is procured. Of course the pastry is utterly unfit to eat.

The bread is poor, sour and wretched; it is whitened chemically, after the life of the grain has been sifted out. With infinite pains it has been rendered worthless.

The milk is, on the average, blue, watery and ill-flavored. In fact, there is absolutely nothing fit to eat, nothing fresh, palatable and nutritious.

In New York some attempts have been made at reform. Some new places have been established, under new names. "Quaker Dairies" have been instituted, the title speaking of simple viands and purity. How completely the practice has sustained the name, we are unable to assert; but we can speak a good word for some of these places. One of them—the original one, we believe—situated next door below Grace Church, on Broadway, was established by the editor of a daily newspaper here, and the plan has been to make it a safe place of resort for the tired and hungry who seek wholesome refreshment. So far as we are able to judge it is all of this. Care is taken to provide pure foods, and healthful foods. The cereals receive attention, and the milk seems to be undiluted and fresh. We do not know the proprietor, but we often sit at his table and enjoy the excellent oatmeal mush or Boston brown-bread toast, which is so palatably served here. The nuisance of tobacco fumes does not assail us, nor does the oppressive odor of stale beer. The place is sweet and cleanly, and the attendants are watchful and kindly. If this place of entertainment is not all we would have it, it is at least the best we have visited, for those who would live wisely and well, and we have no doubt that time and experience will work many improvements, even here. There is one addition which he must make to his bill of fare, or it will remain incomplete. He must supply his customers with the Granulated Wheat Biscuit, now being largely sold by the Health Food Company, at 137 Eighth street, New York. This article would be appreciated by the intelligent people who frequent the Quaker Dairy, and it would, when known, become the most popular bread on the bill, as it surely would prove the most wholesome and nutritious.

